# Editorial: New Trends in Table Olive Fermentation

**DOI:** 10.3389/fmicb.2019.01880

**Published:** 2019-08-13

**Authors:** Joaquin Bautista-Gallego, Francisco N. Arroyo-López, Albert Bordons, Rufino Jiménez-Díaz

**Affiliations:** ^1^Food Biotechnology Department, Instituto de la Grasa, CSIC, University Campus Pablo de Olavide, Seville, Spain; ^2^Departament de Bioquímica i Biotecnologia, Facultat d'Enologia, Universitat Rovira i Virgili, Tarragona, Spain

**Keywords:** table olives, biofilms, lactic acid bacteria, yeast, biotechnological applications, omics

Table olives are a traditional food of the Mediterranean Basin with many centuries of history. They are prepared with fruits obtained from cultivated *Olea europaea* subsp. *europaea* var. *europaea* trees and their production has been also expanded for many countries all over the world (South and North America, Australia, etc.). At present, table olives have an average annual worldwide production of 2.7 million tons (International Olive Oil Council (IOC), 2018), representing an important economic source in the producing countries.

This fermented vegetable has several interesting characteristics. From one side, table olives contain several attractive nutritional compounds such as monounsaturated and polyunsaturated fat, fiber, vitamins, or minerals; and from the other they have important concentrations of well-known bioactive compounds (Garrido-Fernández et al., 1997; Preedy and Watson, 2010). Thus, these properties have led scientists to propose table olives as a functional food. However, there are some drawbacks which have limited the use of this claim: (i) the high caloric content (ii) the sodium level (5–8%, except black olives by oxidation with 2% NaCl). However, because of the high presence of other healthy compounds, the olives could be considered as a healthy food, given the usual composition and proportion of some of its components (oleic acid, vitamin E, vitamin A, calcium, maslinic acid or hydroxytyrosol, among others). Moreover, table olives can be carrier of beneficial microorganisms to consumers (Peres et al., 2012), which is another exciting property from the functional point of view.

Finally, one of the most important challenge of the industry is the treatment of effluents generated during olive processing, because of their high volume of production, conductivity, and chemical oxygen demand (COD), especially in countries with scarce water resources.

The Research Topic “New Trends in Table Olive Fermentation” belongs to the Food Microbiology section in the Frontiers in Microbiology journal. It covers a total of 15 contributions divided in two reviews and 13 original research papers. Many of the most relevant researchers in the field have collaborated in its elaboration.

We present an overview of these papers which can be grouped under different research themes as follows: (i) microbial stability and safety conditions; (ii) table olives as carrier and source of probiotic lactic acid bacteria (LAB); (iii) the effect of new procedures on the sensory profile of table olives; and (iv) the management of table olive wastewater. The diversity of research displayed in this Research Topic demonstrates the important potential of this product and its impact in the fermented vegetables economy.

In the first group of papers, Perpetuini et al. have evaluated the impact of different irrigation regimes on the fermentation process of inoculated and spontaneous *Itrana* table olives. At harvest and fermentation, the concentration of phenolic compounds was always higher in olives from trees which had received less water. Furthermore, the use of a LAB starter positively influenced the fermentation process whereas the irrigation regime did not alter the microbial dynamics or brine acidification.

Bavaro et al. have described different molds isolated from Italian and Greek fermented black table olives. They carried out a technological and safety characterization and determined different strains belonging to *Penicillium roqueforti* (4) and *P. panem* (1) which could be used as co-starters with LAB and yeasts. Also, in black olives, Bonatsou et al. aimed at the yeast diversity during the *Kalamata* natural fermentation under different initial acidification procedures. The dominant yeast species at the beginning were *Aureobasidium pullulans* for control and acidified treatments, and *Candida naeodendra* for lactic acid treatment. At the end of the fermentation, the dominant species were *Candda boidinii* and *Candda molendinolei*. Thus, the different acidification agents, such as vinegar and lactic acid, affected the final composition of yeast species on olives.

Rodríguez-Gómez et al. have studied a high value green table olive variety *Aloreña de Málaga*, which has been the first variety in Spain under Protected Designation of Origin (PDO) recognized by the European Union (DOUE, 2012). Authors focused in the effects of a previous heat shock treatment on the fermentation and packing processes. Interestingly, the application of this treatment was beneficial since it favored the LAB growth, a better green appearance color of fruits and improved the stability of the packaged product. Regarding the same variety, Ruiz Bellido et al. deepened in the lack of standardization of production processes and HACCP systems. Authors based a decision-making scoring system on the identification of potential hazards or deficiencies in hygienic processes for the subsequent implementation of corrective measures to standardize production process. They found that corrective measures should be focused on reducing the microbial contamination of brines and fruits at primary steps and the implementation of novel treatments on olive dressings (irradiation, scalding, and ozonation). Furthermore, industry could reduce the levels of salt and preservatives in packaging producing a healthier product.

Regarding to the safety issues, Bevilacqua et al. have studied the effect of sugar, NaCl and temperature on the survival of *Staphylococcus aureus* and *Listeria monocytogenes* in synthetic brine. Thus, the addition of sugar (a widespread practice in producers) could be a challenge as it could increase the survival of some pathogens. In addition, Lavermicocca et al. delved in the bio-preservation of *Bella di Cerignola* table olives during refrigerated storage. Authors used *Lactobacillus plantarum* 5BG and showed a better performance by halving brining volumes and avoiding chemical stabilizers, and significantly reducing the salt concentration. In addition, the final product was also safely preserved for almost 5 months as suggested by the reduction of the survival rate of *L. monocytogenes*.

In their review, Campus et al. focused on current technologies and recent advances in the processing technology of table olives. They proposed the use of some pre-processing technologies (ionizing radiations, ultrasounds and electrolyzed water solutions), the use of LAB starter cultures, salt reduction strategies, table olives as carrier of probiotic LAB and new packaging technologies (modified atmosphere, high pressure processing and biopreservation).

With concern of table olives as source and carrier of probiotic lactic acid bacteria, four original researches overview different studies to their implementation. Guantario et al. have provided a first approach of the characterization and probiotic potential of LAB strains isolated from *Nocellara del Belice* table olives. One *Lactobacillus pentosus* and one *L. coryniformis* strains significantly induced prolongevity effects (*in vivo* study with the *Caenorhabditis elegans* model), protection from pathogen-mediated infection, adhesion to human intestinal epithelial Caco-2 cells and were able to outcompete foodborne pathogens for cell adhesion.

Pino et al. delved the fermentation of *Nocellara Etnea* table olives at low salt levels by the probiotic starter *Lactobacillus paracasei* N24, focusing on the volatile organic compounds (VOCs) formation. Even more, the starter was persistent until the end of the fermentation, revealing its promising perspectives as starter culture. Both microbial population and VOCs were slightly affected by salt content while a strong influence was determined by time of fermentation.

Rodríguez-Gómez et al. and have studied the factors that may affect the survival of a probiotic starter in large-scale fermentations of green Spanish-style olives. Authors proposed three important recommendations: inoculation after brining to reduce the presence of initial wild microorganisms, the use of re-inoculation to replace the possible initial died starter, and the starter survival is higher at the beginning of the season.

Abriouel et al. emphasized on the importance of the genetic base for the health promoting capacities of *L. pentosus* MP-10, isolated from *Aloreña de Málaga* table olives. Authors carried out *in silico* analysis of this strain's carbohydrate metabolism (as glycoside hydrolases, glycoside transferases, and isomerases) and the proteins (as mucus-binding) involved in the interaction with host tissues.

However, all modifications and starters used during table olive fermentations can alter the final product so researchers have to control their possible effect on the sensory profile. López-López et al. have evaluated the organoleptic profile by 200 consumers of traditional spontaneously fermented and potentially probiotic (inoculation of *L. pentosus* TOMC-LAB2) green table olives. Both final products were perceived similarly and they detected that the attributes with favorable influence on the *Overall score* and the *Buying predisposition* were *Appearance, Odor*, and *Crispness*; on the contrary, *Salty* had a marked adverse effect. Bautista-Gallego et al. have investigated the influence of the addition of zinc chloride in packaged natural black table olives. The presence of this salt in the packaging brine reduced the sugar diffusion and the bitter perception, while increased the overall acceptability, hardness and crunchiness.

Finally, Rincón-Llorente et al. have reviewed the problematic high quantity of wastewater produced by the table olive industry. At present, there is no standard treatment for these wastewaters with acceptable results. Twadahe most common treatment is the storage of wastewaters in large evaporation ponds where disappear due to evaporation. However, this treatment also has a high number of problems (bad odors, insect proliferation, and the contamination of underground aquifers). Other alternative wastewater treatments could be ozonation, Fenton's reaction, TiO_2_ photocatalysis, electro-coagulation, biological treatments (anaerobic or aerobic), and bioremediation technologies, among others. A promising alternative would be an integrated purification processes combining a first step of chemical oxidation (i.e., electrochemical treatments with boron-doped diamond), with a second biological step.

The varied contributions to this Research Topic are evidence of the study undertaken by researchers that provide an updated and high-quality overview of the current work on the table olive fermentation. We hope that this Research Topic informs readers properly about the benefit of this product and the challenges that have yet to be overcome in this field.

## Author Contributions

All authors listed have made a substantial, direct and intellectual contribution to the work, and approved it for publication.

### Conflict of Interest Statement

The authors declare that the research was conducted in the absence of any commercial or financial relationships that could be construed as a potential conflict of interest.
